# Impact of heteroaggregation between microplastics and algae on particle vertical transport

**DOI:** 10.1038/s44221-024-00248-z

**Published:** 2024-06-13

**Authors:** Francesco Parrella, Stefano Brizzolara, Markus Holzner, Denise M. Mitrano

**Affiliations:** 1 Department of Environmental Systems Science, ETH Zürich, Zurich, Switzerland; 2 Institute of Environmental Engineering, ETH Zürich, Zurich, Switzerland; 3 Biodiversity and Conservation Biology, WSL, Birmensdorf, Switzerland; 4 Environmental Microbiology, EAWAG, Dübendorf, Switzerland; 5 Institute of Hydraulic Engineering and River Research (IWA), University of Natural Resources and Life Sciences, Vienna, Austria

**Keywords:** Environmental impact, Biogeochemistry, Hydrology

## Abstract

Understanding the impacts of microplastics (MPs) on aqueous environments requires understanding their transport dynamics and how their presence affects other natural processes and cycles. In this context, one aspect to consider is how MPs interact with freshwater snow (FWS), a mixture of algae and natural particles. FWS is one of the primary drivers of the flux of organic matter from the water surface to the bottom sediment, where zooplankton, diurnal migration, fish faecal pellets settling and turbulent mixing can also play prominent roles. Understanding how MPs and FWS heteroaggregation affects their respective settling velocities is important to assess not only MPs fate and transport but also their ecological impacts by altering FWS deposition and thereby nutrient cycling. In this present study, we obtained a mechanistic understanding of the processes controlling MPs settling dynamics and heteroaggregation with FWS and the subsequent impacts on the settling rates of both MPs and ballasted FWS. Here we used a plexiglass column equipped with a stereoscopic camera system to track the settling velocities of (1) MPs of various compositions, densities and morphologies, (2) FWS flocs and (3) MP–FWS agglomerates. For each experimental set, thousands of particles were tracked over a series of image sequences. We found that agglomerates with high-density MPs settled at least twofold faster than FWS alone, implying a much smaller residence time in the water column, except for cases with MP fibres or low-density plastics. These findings will help to refine MP fate models and, while contingent on MPs number, may impact biogeochemical cycles by changing the flux of nutrients contained in FWS to the sediment.

## Abstract (web summary)

The interactions between microplastics and freshwater snow can influence the way in which both particle types settle in freshwater environments. Advanced and automated tracking techniques show that agglomerates of the two particles settle faster than the individual components alone, underscoring the potential repercussions on biogeochemical cycles.

## Main

In aqueous environments, the flocculation of natural particles such as clays, biomacromolecules and microorganisms (phytoplankton or algae) leads to the formation of flocs, also known as marine snow (MS) or freshwater snow (FWS), depending on the water body. They are formed by collision of particles suspended in water depending on the degree of shear forces and ionic strength^1–3^. Although determining exact snow abundances is challenging, especially when considering heterogeneity across large water bodies, their widespread occurrence has been largely reported. This is true for both marine environments^1,4^ and, while fewer data are available, for freshwater bodies^2^. Reported concentrations vary widely, from relatively low levels in clear oligotrophic waters to much higher concentrations in areas with abundant biological activity and nutrient inputs. As the flocs become larger, they sink from the water surface to the bottom sediment, along the way incorporating more suspended organic and inorganic particles while also acting as a food source throughout the water column^5^. Reported settling velocities of MS and FWS showed large variations depending on their composition, which varies with water chemistry, turbulence and particle characteristics^1,6,7^. In some cases, larger aggregates (4–5 mm) settled slower than smaller ones (1–2 mm) due to their higher morphological complexity and decreased density as size increased^8^. Alternatively, flocs can settle faster as size increases when they incorporate more dense materials as ballast into their structure. MS and FWS settling rates can be difficult to measure in laboratory settings due to sampling and transport issues which cause flocs to dry out and break apart^8^. Accurately investigating the sinking rate of MS and FWS agglomerates under controlled laboratory conditions is crucial^9^ for understanding their formation and vertical transport through the water column to assess nutrient fluxes^10^.

Physical fragmentation of large macroplastic litter can result in microplastics (MPs <5 mm) formation, which are widespread in both marine and freshwater environments and include a broad distribution of particle sizes, polymer compositions and morphologies^11^. These physiochemical differences can influence their settling velocity and the potential for heteroaggregation with natural particles, but constraining how these parameters influence MPsʼ fate has proven challenging to date. Although much has been discussed about MPsʼ settling dynamics in relation to biofouling^10,12–14^, their interactions with biofilms substantially differs from heteroaggregation of MS and FWS. The growth rate of biofilms is influenced by the polymer surface chemistry and the surface area to volume ratio^12,15^. As progressively thicker biofilm layers are formed on MP surfaces, the overall mass of MPs increases^16^, expediting their deposition rate towards the sediment. Conversely, MPs may be directly incorporated into FWS through the collision of algae and other natural particles under turbulence, although the collision rate and subsequent number of MPs included in FWS will vary depending on relative concentrations of each particle type.

It is uncertain how well existing models estimate particle settling dynamics in water based on Stokes’ law, which predominately is applied to spherical particles. This is especially true when MPs deviate from spherical shapes such as fragments or fibres or when they form agglomerates with natural particles. Previous studies have assessed only a limited number of particle morphologies, such as spheres and cylinders^17^, and have not considered heteroaggregation^17,18^, resulting in a notable knowledge gap that hampers robust assessment of MPs settling rates. Consequently, to unravel the settling velocities of MPs, a new approach is needed including (1) tracking considerably more (individual) particles (MPs and FWS) in controlled laboratory settings, (2) MPs interactions with other natural particles and (3) more accurate and automated experimental designs to accelerate data collection and processing to measure particle dynamics with higher precision.

**Fig. 1:**
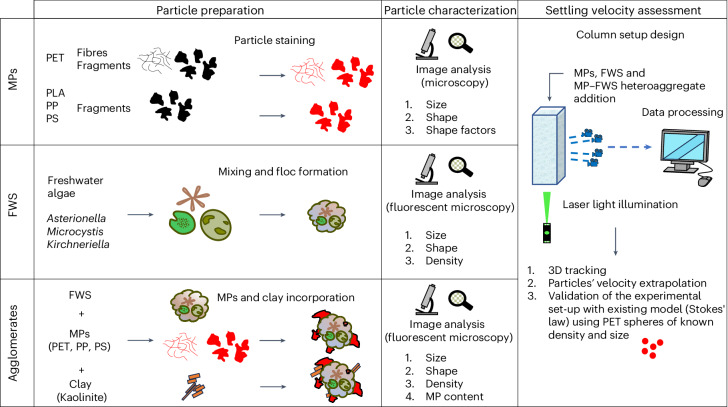
Schematic of the overall experimental design. The overarching experimental design consisted of four steps, where (1) MPs of various sizes, densities and morphologies were characterized with microscopy and stained with fluorescent dye for easier visual identification by cameras, (2) FWS was artificially produced in laboratory conditions by mixing freshwater algae on a roller table and subsequently characterized with microscopy (for shape and size) and density, (3) MPs and FWS agglomerates were formed and characterized and (4) all particles were spiked into a laser illuminated plexiglass column and their velocity tracked with a camera array to measure the settling of thousands of particles per experimental run.

In this context, the heteroaggregation between MPs and FWS is a key consideration for the fate and transport of MPs and an important aspect that could change the cycling of organic matter and nutrients through the water column by altering the settling rate of FWS when MPs are included as a ballast^19–22^. In this study, we provide fundamental insights on how MPs settle in water systems by understanding the dependencies of particle size, density and morphology while also quantifying changes in MP settling rates when acting as ballasts incorporated into FWS (Fig. 1). While we specifically highlight freshwater ecosystems and FWS here, the results and methods are also applicable to marine environments, as the snow flocs have similar behaviour and morphologies across water bodies^2,23^. A column set-up was designed to track thousands of particles per experimental run, and calculation of the settling velocities were performed using the software openPTV for accurate and streamlined analysis. The experimental design was validated by ensuring spherical particles of a known density and size had the same measured velocity as predicted by Stokes’ law. Next, FWS flocs and FWS–MPs agglomerates were created in laboratory conditions, characterized in terms of size and density and their settling velocities were assessed. A comparison between all model particles allowed us to understand variations of the settling velocities of individual MPs and FWS and their agglomerates. Collectively, we provide key insights on the settling rates of MPs, which can be used as input values for fate models in freshwater (and marine) systems^24–26^ and highlighting the potential of how MPs act as ballasts in FWS, which may impact biogeochemical cycles through altering FWS settling in natural waters.

## Characterization of MPs and formation of FWS

Polyethylene terephthalate (PET, 1.40 g cm^−^^3^), polylactic acid (PLA, 1.25 g cm^−^^3^), polypropylene (PP, 0.90 g cm^−^^3^) and polystyrene (PS, 1.04 g cm^−^^3^) white fragments and PET fibres were produced by cryo-milling pre-production polymer pellets or extruding PET filaments, and all MPs produced and tested in this study had highly irregular shapes in the three size classes (<63, 63–125 and 125–250 μm) (Supplementary Fig. 1).

It is important that realistic model MPs are used in laboratory-based studies to better approximate and understand environmental fate and behaviour^27^. The incorporation of MPs into FWS is subject to several variables, including MPs size, density and surface chemistry. For the latter, it has been suggested that different polymer chemistries influence the propensity of agglomeration between MPs and algae^28^. In this context, the formation of an eco-corona can influence how MPs behave in aquatic environments. While the composition of the eco-corona will vary depending on water chemistry parameters (that is, biomacromolecule composition such as differences in wastewater treatment effluent, natural organic matter and so on), the presence of an eco-corona may mask initial polymer surface chemistries effectively making them more similar^29^. Consequently, the aggregation behaviour observed in this study, where the eco-corona consisted mainly of EPS, may be similar to the overall behaviour in other environmental contexts even if it is composed of different biomacromolecules.

The size of FWS flocs produced ranged between approximately 200 and 2,000 μm, and their shape varied from almost regular ellipsoids to strongly irregular (Supplementary Fig. 2). Although the concentration of algae cells was kept constant between each experimental replicate, the formation of FWS was stochastic and consequently the number of flocs produced, and their characteristics, were variable. While there are no standardized methods to create FWS in laboratory conditions, we adapted a number of studies replicating marine snow as a basis for realistic FWS formation^9,30^. Nevertheless, other environmental conditions may alter the resulting flocs in terms of size or morphology, including algae composition, ballasts, pH, temperature and particle collision rates under turbulence.

## Formation of agglomerates depends on MP physicochemical properties

MP–FWS agglomerates formation was a stochastic process resulting in particles that had both a wide size distribution and variable shapes. No statistically significant differences were measured between the size of FWS alone and PET agglomerates (equivalent spherical diameter (ESD) range 100–1,600 μm), whereas PP and PS agglomerates (ESD range 200–1,000 μm) resulted in statistically smaller agglomerates than FWS alone (Supplementary Table 1). When MPs were added to the algae mixture to form agglomerates, incorporation of MPs was dependent on their polymer chemistry, size and morphology. Quantitative assessments of MPs:algae ratios in each flock were difficult to achieve, but qualitatively, low-density polymers (PP, PS) were less prone to be incorporated into FWS than PET. This is in line with the hypothesis of Lagarde et el.^28^, that not all MPs will form agglomerates due to differences in surface characteristics of the polymers, such as wettability, which is higher for PET compared with PS and PP. Smaller-sized MPs formed agglomerates more readily than larger-sized particles, and qualitatively, there was a higher MPs:algae ratio within each floc as particle size decreased (Supplementary Fig. 3). Heteroaggregation of MPs and algae resulted in denser flocs than FWS for all polymers, including those that are low density (PP, PS (Supplementary Table 1)). The presence of MPs in the flocs probably reduced the porosity leading to more compacted particles^28^, with the only exception being agglomerates with PET fibres. In these instances, the morphology was a network-like, permeable structure (Supplementary Fig. 3). Despite the presence of large voids in the agglomerates with PET fibres, their estimated density was slightly higher than that of FWS, presumably because of the contribution of the relatively heavier fibres contributing to increasing the average density.

A variant protocol for the formation of artificial FWS and agglomerates included clay particles, which showed a high propensity to be incorporated as a ballast (Supplementary Fig. 4). The overall size, shape and morphology of the resulting agglomerates did not differ substantially from those made only with FWS and MPs. Additionally, sand particles were tested to incorporate different and more dense ballasts into the flocs; the process failed to result in the incorporation of sand as all particles instead settled to the bottom of the vial. Most likely, the sand did not remain suspended long enough to be incorporated into the flocs, even under constant turbulence.

## Validation of column set-up and experimental design

**Fig. 2:**
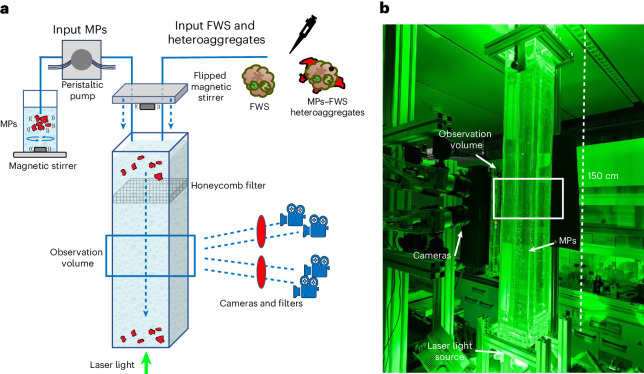
Schematic of the experimental design. **a**, Schematic of experimental design with the different input systems according to the particle considered and camera arrays calibrated on the observation volume. **b**, Photograph of laboratory set-up during a test run using MPs to measure settling velocities.

**Fig. 3:**
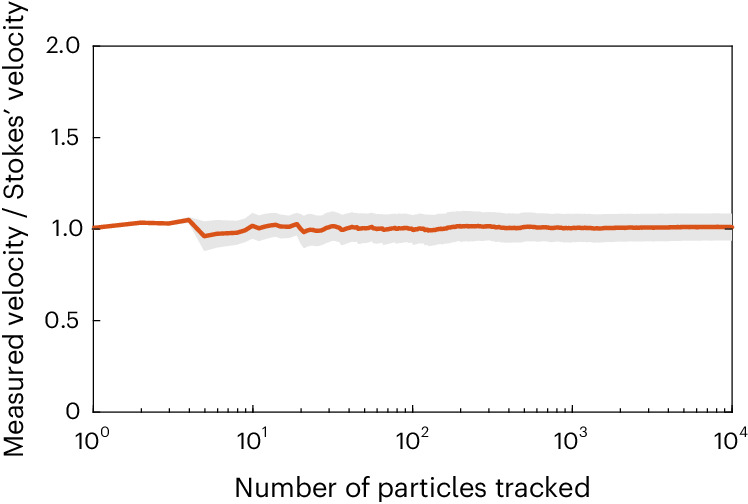
Velocity profile of experimental set-up validation. Assessment of the column set-up, particle tracking, image acquisition and data postprocessing. Spherical fluorescent PET particles (Cospheric, 1,212 kg m^−^^3^, 220–250 μm) were used for the test and the measured settling velocity was then compared with the theoretical settling velocity calculated by Stokes’ law. *Y* axis: ratio between the mean measured particle settling velocity and the predicted Stokes’ law settling velocity. *X* axis (log scale): number of tracked particles whose mean velocity values are considered for the calculation. The red line indicates the convergence profile of the velocity ratio, whereas the shaded area indicates the standard deviation.

A 1.5-m-high plexiglass column, illuminated by a laser and filled with synthetic freshwater, was used for settling experiments (Fig. 2), where MPs, FWS or aggregates were added. A system with four cameras was used to track up to several thousands of individual particle trajectories, providing robust data to measure settling velocities. Further information on the experimental design can be found in Methods. To assess the validity of our experimental set-up in terms of particle dispersion, image acquisition and data processing, the measured settling velocity of model spherical PET particles of known size and density was compared with predicted settling velocities using Stokes’ law. This ratio was assessed to check the convergence profile of the particles’ settling velocities (Fig. 3). The velocity ratio profile converged to a value of 1.01 ± 0.03 after 100 measured particles. The small bias of the average value may be partly attributed to the slight polydispersity of the particle size distribution (Stokes’ law is quadratic in the diameter implying higher weight of larger particles in the average settling velocity) and partly to the finite accuracy of the particle tracking system (the relative velocity error is on the order of 2–3%) (ref. ^31^). These results indicated that the measured velocity was in line with calculations from Stokes’ law and that our experimental set-up was appropriate in determining settling velocities of particles.

The performances of the experimental design presented here made it possible to obtain large amounts of data for each variant of the experiment in a reproducible fashion. The use of four cameras synchronized and calibrated on the observation volume improved data collection and tracked a larger number of model particles (MPs, FWS, agglomerates), while reconstructing their individual 3D trajectories. A more detailed review on the overall performances of the experimental design is available in Supplementary Information, ‘Improved experimental design allowed for wider tracking’.

## Settling velocities of MPs and FWS alone do not predict agglomerates well

**Fig. 4:**
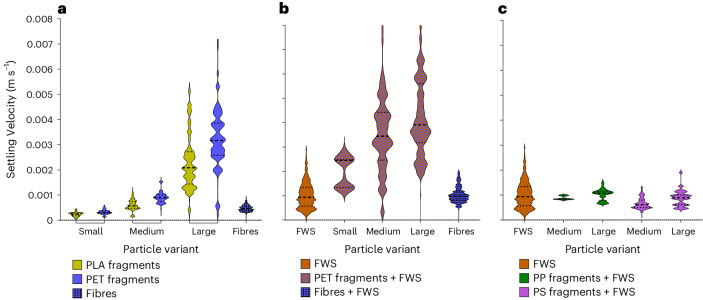
Settling velocities of model particles. **a**–**c**, Violin plots of the settling rates of all particle variants including high-density MPs (PET, PLA) of all size classes and morphologies (fragments and fibres) (**a**), FWS and PET agglomerates (fragments and fibres) (**b**) and FWS and low-density MPs fragments (PP and PS) (**c**). The total number of particles measured for each experimental variant ranged between 10^3^ and 10^5^.

MPs size, polymer density and morphology strongly influenced settling dynamics (Fig. 4). Comparatively, FWS flocs had relatively slow(er) settling velocities despite their large(r) size compared with MPs. Incorporation of MPs altered the settling velocity of FWS depending on the size, density and number of MPs included in each FWS agglomerate. Collectively, this provides a clearer picture of how heteroaggregation influences MPs settling dynamics and how MPs act as ballast impacting the settling rate of FWS and subsequently may alter cycling of nutrients throughout the water column.

### Irregular MP settling dynamics deviate from common models

Across all MP fragments, as anticipated, density and size influenced the settling velocity of particles where mean settling velocities increased for larger and denser particles (Fig. 4a and Supplementary Table 2). For both the largest PET and PLA fragments, there was a wider range of settling velocities compared with the other size classes, which is consistent with the larger size distribution of the primary particle sizes used in the experiments (Supplementary Fig. 5 and Supplementary Table 3). Individual PP and PS MPs were buoyant and did not settle. PET fragments and fibres exhibited reduced settling velocities when compared with previous investigations involving MPs of similar shape, as shown in Table 1 ref. ^17,32^. However, here larger (and sometimes denser) MPs were used, resulting in higher settling velocities. MPs of all sizes are found in aqueous environments, and smaller particle sizes are often found at higher concentrations^33–35^. Therefore, investigating smaller MP sizes is important, especially when considering the lack of data currently available. Our experimental design enabled us to accurately assess the velocities of these smaller, irregular-shaped MP fragments, yielding precise velocity results to fill in this knowledge gap.

**Table 1:** Comparison of settling rates for MPs, FWS and MS

Particle type	Settling rate (m s^−1^)	Size (mm)	Reference
MPs (PET, PLA) irregular fragments MPs (PET) fibres	2.39–32 × 10^−4^ 4.78 × 10^−4^	0.063–0.250 0.5	Current study
MPs (PET, PVC, PS) irregular fragments MPs (PET, CoPA) fibres	19–1,200 × 10^−4^ 10–700 × 10^−4^	0.4–5 5–15	Waldschläger and Schüttrumpf^32^
MPs (PCL) cylinders MPs (fishing lines, 1,130–1,168 kg m^−^^3^) fibres	597 ± 208 × 10^−4^ 67–246 × 10^−4^	0.59–6.23 0.15–0.71	Khatmullina and Isachenko^17^
MPs (PET) irregular fragments	116.8–1,129.1 × 10^−4^	0.3–2.5	Wang et al.^36^
FWS Artificial flocs of diatoms, green algae, cyanobacteria	10.3 ± 6 × 10^−4^	1.161 ± 0.35	Current study
FWS Natural flocs of Daphnia moults	4.29 ± 0.2 × 10^−4^	3	Grossart and Simon^23^
FWS Natural flocs of Copepods carcass	6.59 × 10^−4^	3	Grossart and Simon^23^
FWS Natural mixture of diatoms, green algae, cyanobacteria	2.9 ± 0.1 × 10^−4^	6–12	Grossart and Simon^23^
FWS and MPs Agglomerates of natural freshwater and PP	3	0.125–2	Semcesen and Wells^15^
MS Natural flocs, in situ and in laboratory measurements	8.56 ± 4.5	2.4–75	Alldredge and Gotschalk^6^
MS Natural flocs	3–15 × 10^−4^	0.01–0.1	Hawley^57^
MS Natural flocs, in situ measurement	4 × 10^−4^	1–5	Asper^8^
MS Natural flocs, in situ measurement	1.96–30 × 10^−4^		Kajihara^58^
MS Natural flocs, in situ measurement	11–17 × 10^−4^	1–12	Lampitt^59^
MS Natural flocs, in situ measurement	4.9–10 × 10^−4^	2–11	Shanks and Trent^9^
MS Mixture of diatoms or coccolithophorids	5.9–84	0.1–2.9	Ploug and Inversen^37^
MS Mixture of diatoms and cryptophyte	23 × 10^−4^	1.8–3.5	Long et al.^20^
MS Artificial flocs	39–115 × 10^−4^	2–6	Prairie et al.^30^

Values are either reported as an average size with standard deviation (±) or with the approximate size range, as per the initial publication.

**Fig. 5:**
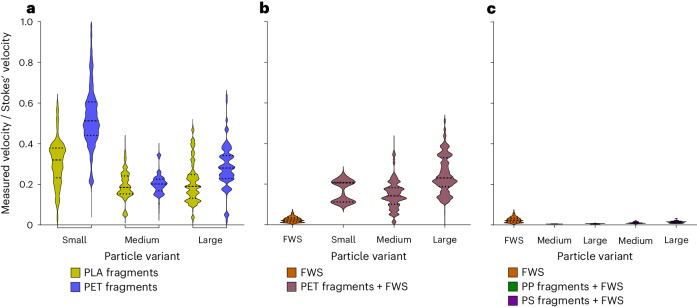
Ratio of measured and Stokes’ settling velocity for all model particles. Violin plots of the ratio between the measured velocities from the experimental data and the respective Stokes’ law prediction. The Stokes’ velocity was determined based on the effective density and size of the particles, approximating a circular shape. **a**, Velocity ratio of individual MPs with density higher than water (PLA, PET) of different size classes. **b**, Velocity ratio of FWS and PET fragments agglomerates. **c**, Velocity ratio of FWS, PP agglomerates and PS agglomerates of different size classes. Incorporation of MPs into FWS lead to larger deviations from the Stokes’ model.

By comparing the measured velocities of irregular MPs to ideal settling velocities of spherical particles of the same size and density using Stokes’ law, we can appreciate how strongly irregular shape influenced particle settling rates (Fig. 5). For all MP fragments, settling velocities were lower than ideal spherical particles, which is consistent with previous studies^36^. Consequently, due to increased drag resistance, irregular MPs will stay in suspension for longer time periods. However, the deviation from Stokes’ law varied with particle size and polymer density, which was correlated with the extent of shape irregularity among the MP variants. The circularity of the fragments (Supplementary Fig. 1) was not constant across MP size classes and was slightly higher for the small fraction of PET compared with larger particle size, meaning that despite all fragments having an irregular shape (Supplementary Fig. 6), the smaller-sized particles were more spherical. This is probably an artefact of how these model MPs were produced, and we would anticipate that environmental MPs of all sizes could have varying degrees of irregularity regardless of particle size. Notably, PET fibres fell notably slower than the medium- and large-sized fragments, despite similar masses. This can be attributed to their tendency to orient horizontally with respect to the settling direction, which increased their drag resistance. Collectively these findings indicate that MP shape irregularity is the key parameter in settling rate deviation from Stokes’ law.

### Settling velocities increase after aggregation of MP–FWS

Despite their markedly larger mean size compared with MPs, FWS flocs did not always settle faster (Fig. 4b). The settling rates for FWS measured here were consistent with previous studies of similar size range^8,37^. Considering that the FWS flocs produced in this study were generally smaller in size than both natural and artificial ones from other works (Table 1), our results align with Asper et al.^8^, who hypothesized that small flocs have a lower porosity than larger ones, which makes them denser and thus increases their settling velocity.

Agglomerates with PET MPs fragments settled faster than individual MPs or FWS alone, and settling velocities increased with the size of the MPs in the agglomerates (Fig. 4b). As the size distribution (and settling velocities) of MPs in each size fraction were relatively large, this also influenced the distribution of agglomerates settling, which had a wider distribution than for FWS alone. In contrast to PET fragments, when less dense MPs (PP, PS) were incorporated into agglomerates, the settling velocity of FWS remained unchanged no matter the MP size (Fig. 4c). The settling velocity of agglomerates with PET fibres did not considerably differ from FWS but was higher than individual fibres. FWS and each variant of PET agglomerates settling velocities showed an even higher deviation from Stokes’ law predictions compared with the respective individual PET MPs (Fig. 5). The main reason was the morphology and porosity of the agglomerates, which are factors not directly considered in the Stokes’ model but change the drag resistance of particles. Similar to MPs, the agglomerates were irregularly shaped, which further contributed to the deviation from the Stokes’ prediction.

**Fig. 6:**
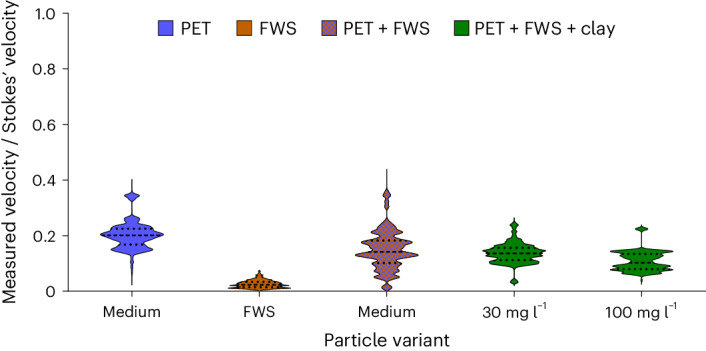
Ratio of measured velocity of agglomerates consisting of FWS, PET MPs and clay and Stokes’ model. Violin plots of the ratio between the measured velocities and the respective Stokes’ law predictions for the following groups of particles: PET MPs fragments, FWS flocs, agglomerate FWS + PET MPs (size 63–125 μm) and agglomerate FWS + PET MPs + clay at two concentrations in suspension. The Stokes’ velocity was determined based on the effective density and size of the particles, approximating a circular shape. The normalized settling velocities of the agglomerates upon incorporation of clay as ballast material into the FWS was similar to agglomerates with only FWS and MPs.

The presence of other ballasts in the FWS, such as clay, could influence the settling dynamics in a few ways. First, the inclusion of denser ballasts could augment the particles’ mass, subsequently resulting in an increased settling velocity. Second, the introduction of particles might cause alterations in the flocs’ morphology, including filling small voids leading to subsequent changes in settling velocity^20,38^. Because of this, we additionally assessed the normalized settling velocity for agglomerates containing clay. Although we could not visually observe structural changes in the FWS using microscopy (Supplementary Fig. 4), the introduction of clay may have potentially filled voids, compacting the floc and thereby increasing the settling velocity due to decreased drag resistance. To investigate these hypotheses, we compared the normalized settling rates for three types of agglomerate: FWS alone, FWS combined with MPs and FWS along with MPs and clay at two different clay suspension concentrations (Fig. 6).

The normalized falling velocity of agglomerates with clay closely resembled those composed of only FWS–MPs, indicating that an insufficient number of clay particles were included to consider it an effective ballast under these conditions. With regards to the increase in mass, the size of the clay particles is much smaller than the MPs tested here, and so a much larger number of particles (as quantified in the following section) would have to be incorporated into the floc to result in a comparable contribution to the increase in settling velocity. Additionally, clay may not have appreciably filled floc pores, and thus the settling velocity remained similar to other MP–FWS agglomerates. Nevertheless, it must be noted that a number of key variables in these experimental settings represent a fraction of the diversity that exists in environmental water compositions (for example, clay mineral type, concentration, size, salinity), and therefore in some cases small mineral particles could effectively contribute as ballasts to flocs in some settlings.

### Agglomerate settling velocity depends on particles traits

The overall density of PET agglomerates were lower compared with PET MPs alone, and it could be expected that this would reduce the falling velocity of PET agglomerates. However, this was not the case, probably due to the much larger size of the agglomerates (up to 1,600 μm) compared with the individual MPs. Therefore, the size of the agglomerates had a dominant impact on the settling dynamics of particles. Compared with individual PET MPs, algae aggregation reduced the overall density of the final agglomerates but increased their radius, which overcame the density change, and consequently the sinking velocity increased.

Whereas PET agglomerates and FWS had similar ESD distributions, the density of the agglomerates were larger than FWS alone, leading to considerably faster settling velocities. Therefore, the difference in density between PET agglomerates and FWS was the driving factor for the increased settling rate, despite similar sizes. From the measured velocities of agglomerates, the approximate number of MPs inside a floc could be estimated (Supplementary Information, ‘Estimation of the number of MPs incorporated into FWS flocks’) and qualitatively compared to microscopy images (Supplementary Fig. 3). PET agglomerates settled faster than FWS by a factor of 1.9, 3.5 and 4 for small, medium and large fractions, respectively. From these calculations, the number of MPs per agglomerate was approximately 13, 4 and 1 for the small, medium and large size fractions, respectively. This was qualitatively consistent with the microscopy images, where PET fragments were more prone to form agglomerates than PP or PS, and smaller MPs were more abundant in flocs than larger ones.

Following the calculations used to assess how the inclusions of ballasts influence particle density (Supplementary Information, ‘Estimation of the number of MPs incorporated into FWS flocks’ and equation (5)), we can also confirm that clay is an ineffective ballast material under the scenario studied here. To achieve a similar settling velocity increase as observed for MPs (that is, a 3.5-fold increase for medium-sized PET agglomerates), the incorporation of over 100,000 clay particles would be required assuming a clay density of 2,600 kg m^−^^3^. This difference arises because the number of particles needed for a given velocity increase scales cubically with the diameter, while it scales linearly with density. Although some recent work has suggested the possibility of clay alone to act as a ballast for buoyant MPs without the presence of algae^39^, this was only explored at higher salinities, clay concentrations and in the presence of surfactants. Collectively, the results from both the experimental settling and calculations support the fact that MPs, as dense and larger ballast, have a greater influence on agglomerate mass and settling velocity than clay.

In contrast to PET, the impacts of PP and PS as a ballast did not considerably change the settling velocity of FWS. This could be due to the relatively lower abundance of MPs in the agglomerates compared with agglomerates with PET. Moreover, the difference between the density of individual PP, PS and FWS was smaller than those between PET and FWS. Agglomerates with fibres also had a similar settling velocity to FWS and had a statistically slower settling velocity than agglomerates with PET fragments. This was probably due to the network structure of floc prompted by fibre incorporation compared with fragments, where the fibres increased agglomerate porosity and consequently increased drag resistance and decreased settling velocity.

## MP incorporation into FWS may influence nutrient cycling

MS and FWS flocs are formed near the water’s surface and influence the global nutrient cycle through recirculation and providing macronutrients to organisms throughout the water column^9,40^. However, this is only a portion of the total nutrient flux. Other important drivers of nutrient cycling can include zooplankton diurnal migration, fish fecal pellets settling and turbulent mixing. To understand how MPs could impact these dynamics more fully, one would also need to assess how MPs could impact the settling of other particles (for example, when they are incorporated into fecal pellets) that also contributes to vertical nutrient fluxes. Recent works explored how MPs potentially impact (by up to 60%) key processes of the biological carbon pump, such as zooplankton grazing rates, in a certain oligotrophic zone where MPs were more abundant^41,42^. While MPs will not directly impact nutrient fluxes, they may indirectly contribute in this context because under turbulent mixing, they may become incorporated into FWS. This is precisely the process we aimed to assess in this study. When snow flocs sink from the surface water, they undergo re-mineralization by bacteria. Moreover flocs, enriched in particulate organic carbon, transport carbon to sediment and act as a key carbon sink in deep water^1^. Therefore, alterations of the flocs‘ settling dynamics due to interactions with MPs can influence not only an important food source for microorganisms but also the water carbon-storing capacity, potentially having consequences on the water acidity over longer timescales^43^. In this study, the impact of MPs as a ballast incorporated into flocs increased the settling velocity of the agglomerates by at least a factor of 1.9. Such an enhancement in settling rates would have even stronger implications in marine systems, where the depth of the water column is generally greater than freshwater bodies.

Importantly, in natural aqueous systems, the heteroaggregation between FWS or MS and MPs depends on their respective concentrations. The reported concentration of MPs in water bodies varies geographically and are reported in different units of either particle number or plastics mass but can be up to 1,000 g km^−^^2^ in oceans^35^ and between 4.3 × 10^4^ and 46.6 × 10^4^ particles per km^2^ in freshwater^44,45^. Likewise, the concentrations of FWS and MS fluctuates across different locations and over water depth. For example, Pilskaln et al.^4^ estimated a snow abundance between 0.5 × 10^3^ and 13 × 10^3^ aggregates per m^3^ (ref. ^4^). The experimental conditions used here to study heteroaggregation between MPs and FWS may be an exaggeration of processes occurring in natural systems, where particles are more diluted and the collision frequency is less frequent. In natural settings, these processes unfold across larger time and spatial scales, and even in scenarios where MPs are comparatively more diluted, this does not negate the possibility that MPs will form agglomerates. The aggregate formation mechanisms used here probably enhanced heteroaggregation because of the constrained volume, leading to the inclusion of multiple MPs into each aggregate. Whereas the absolute number of MPs included in individual agglomerates may be higher here than anticipated under natural conditions, it does not change the overall trends we describe, that is, that the inclusion of MPs as a ballast in FWS consistently enhanced their settling velocities. Therefore, this process is still an important factor to consider both for understanding MPs fate and transport and for changes in nutrient cycling by altering FWS and MS settling dynamics due to MPs pollution. While we did not directly assess alterations in biogeochemical cycling in this study, we could envision that when MPs are included into FWS and increases their settling rates, this could be a relevant aspect to consider. Nevertheless, these larger-scale impacts are as of yet still hypothetical and largely dependent on the concentrations of various constituents (MPs, FWS flocs, agglomerate formation) in a given circumstance.

For floc formation, particle collision must occur, and consequently the FWS flocs produced here were exposed to turbulence and experienced shear stress. In this study, mild turbulence was only applied during floc formation, and we did not introduce turbulence during the settling experiments. In natural water bodies, turbulence might also transport both natural particles and MPs into regions of the epilimnion with less turbulence and shear stress, where flocs are likely to form and settle^1^. However, under some scenarios, additional turbulence and shear stresses may induce agglomerate break-up, which in turn would impact the settling velocities of both FWS and MPs. Besides particle settling, the incorporation of MPs into agglomerates may also influence their fate and transport dynamics in other water systems, including rivers, but this remains to be assessed.

## Summary

We proposed an innovative experimental design, which included a column set-up, high-speed cameras and automated image acquisition, which allowed us to reconstruct the 3D trajectories of individual particles and extrapolate the information on their settling velocities over time and space. By measuring a large number of particles for each experimental variant, we have been able to better understand the variability in settling velocities across a range of model MPs and FWS agglomerates. This initial dataset confirms the overarching themes that (1) particles with larger mass have faster settling rates, but particle morphology plays an important role in dictating the absolute rate, (2) the larger the density difference between agglomerates components, the larger the change in settling velocities compared with its individual components and (3) aggregate morphology influences settling rate. We additionally showed that Stokes’ law was not able to describe MPs settling velocities accurately, mainly due to their irregular shape, indicating that simplifications in particle transport calculations would underestimate their residence time in the water column.

We aimed to obtain a mechanistic understanding of the processes controlling MP settling dynamics and heteroaggregation with FWS and the subsequent impacts on changes in both MPs settling rates and how MPs can act as a ballast for FWS. To do this, we deemed the use of model systems and test materials necessary to have more control over the variables. Nevertheless, we appreciate that the results we obtained from these studies are based on model systems that are less complex and variable than natural waters, yet the overarching themes are still relevant. This poses a challenge for future theory and here we provide a dataset to benchmark new model approaches for MP transport. Given that residence time in the water column is largely controlled by the particle sinking velocity, this could impact nutrient cycling and productivity throughout the water column and long-term carbon storage in sediments as a sink. However, these hypothetical impacts on biogeochemical cycling are highly contingent on relative particle numbers in the system. Flocs are far more numerous than MPs, and whereas MPs are likely to increase settling rates of FWS as shown here, it remains to be assessed in which scenarios there would be a large enough number of MPs to alter large-scale environmental processes. Collectively, these results underline the importance of needing to understand how MPs interact with natural systems from an ecological point of view, by not only assessing the presence of MPs in the environment but also their indirect impacts on natural processes.

## Methods

### Microplastics preparation and characterization

Polyethylene terephthalate (PET, 1.40 g cm^−^^3^), polylactic acid (PLA, 1.25 g cm^−^^3^), polypropylene (PP, 0.90 g cm^−^^3^) and polystyrene (PS, 1.04 g cm^−^^3^) white fragments and PET fibres were produced by cryo-milling pre-production polymer pellets or extruding PET filaments^46^, respectively. For PET and PLA, three size classes were obtained from sequential sieving after milling, including <63 μm (small), 63–125 μm (medium) and 125–250 μm (large). PP and PS fragments were obtained in two size classes, 63–125 μm (medium) and 125–250 μm (large). PET fibres (20 μm diameter) were cut to an approximate length of 500 μm using a fibre cutting machine previously designed in house^46^. Both fragments and fibres were further characterized in terms of shape and size distribution with a microscope (Keyence 3D Laser Scanning Microscope; Supplementary Fig. 6), where at least 100 individual MPs were assessed per variant. The images were processed with ImageJ to calculate particle length and width, and this information was used to subsequently derive their projected (2D) surface area, average ESD, shape factors (circularity and aspect ratio) and fitting geometric shapes. Further details of particle characterization can be found in the Supplementary Information, including average ESD of all MPs (Supplementary Table 3), size distribution of fragments (Supplementary Fig. 5) and shape factor calculations (Supplementary Fig. 1).

To ensure easier identification in the column and to exclude dust and non-target particles in suspension from the analysis, both fragments and fibres were stained with Nile Red by modifying existing methods^47^. Nile Red (Chemical Abstracts Service (CAS) number 7385-67-3) was dissolved in methanol at a final concentration of 50 mg l^−1^. Approximately 20–30 mg MPs were added in 20 ml glass vials with 3 ml of Nile Red solution and placed in an incubator for 5 h at 60 °C. MPs were then filtered (filter paper, Macherey-Nagel 615, ∅ 55mm) and rinsed with deionized water. Filters were left on a Petri dish to dry overnight before collecting stained particles, which were then stored as a dry powder in a closed vial at room temperature until further use.

To assess the potential of the dye leaching from the particles when suspended in water, the stained particles were suspended in synthetic freshwater (that is, the test media in the column studies) for 0, 3, 7 and 14 days. Subsequently, they were removed from suspension, placed on a filter and imaged with a fluorescent microscope (LEICA DM6000). Images were acquired keeping acquisition settings (intensity, gain and exposure) constant. No differences in fluorescence intensities were detected, meaning that the staining procedure and detection protocol were suitable to visualize the MPs in the experimental set-up.

### Algae cultures

Three algae strains (*Microcystis aeruginosa, Kirchneriella subcapitata, Asterionella formosa*) collected from Lake Greifen (Switzerland) were grown at 22 °C under cool white light (1,521 lumen) with a 12h/12h light/dark cycle. These strains were chosen as representative organisms from different classes of algae, including green algae, diatoms and cyanobacteria. The cultures were prepared in a Wright’s Chu (WC) freshwater medium following the protocol by Kilham et al.^48^ and grown in plastic tissue-culture flasks. Fresh growth media were inoculated into the cultures every two weeks using sterilized equipment in a laminar flow cabinet. Cell counting was regularly performed to ensure cells concentrations of 10^7^ cell ml^−1^ per each algae strain using a Casy TTC cell counter (Scharfe System).

### FWS and FWS–MP aggregate formation and characterization

FWS flocs were created by adapting methods for MS formation in laboratory conditions^20,49^. Here, 8 ml of each algae strain (10^7^ cell ml^−1^) were mixed in 40 ml glass vials and placed on a roller table at 40 r.p.m. The constant rotation of the vials enhanced the collision frequency between the cells, and the release of extracellular polymeric substances (EPS) into the surrounding media enhanced the formation of larger flocs over time^20,28^. Visible flocs were formed within a week of continuous rotation (Supplementary Fig. 2). The same procedure was used to produce agglomerates of FWS and MPs. Independent of MP size or density, the number of MP particles were kept constant (approximately 500) and each MP variant was added individually into separate glass vials containing the algae mixture (10^7^ cell ml^−1^ for each strain), resulting in a final concentration of approximately 20 MPs per ml of algae solution. Given the variable MP sizes, we endeavoured to spike the same number of MPs into the algae suspensions to create flocs, rather than the same MP mass. While several MPs:algae ratios were initially tested, the final concentrations used here were ideal to form agglomerates within a week of continuous rotation.

The FWS and agglomerates protocol was expanded with the addition of silica-based particles. Sand particles (size ranges 63–125 μm and 125–250 μm) were added into the suspension and rolled together with the algae to increase their potential for interaction and subsequent incorporation into the flocs. However, this protocol did not lead to sand being incorporated into the flocs, and all particles accumulated at the bottom of the vial. Most likely, the sand was too dense and thus did not remain suspended long enough to be incorporated into the flocs, even under constant turbulence. Therefore, the protocol was adjusted and FWS was produced either with the three algae species alone or with the inclusion of clay in some variants of MPs-FWS agglomerates. Considering the relative presence of other silica-based particles in surface waters, another protocol to produce artificial FWS flocs was tested, which included the addition of clay particles (Kaolinite, Sigma Aldrich CAS 1318-74-7) at different concentrations (30 mg l^−1^, 100 mg l^−1^) in suspension with the algae and MP solution. After the incubation, the resulting agglomerates showed a similar size and morphology as those without clay. Clay particles (Supplementary Fig. 4) were visible as black dots and probably were homoaggregated in the floc given the size in the images. This suggests a greater incorporation potential than sand, but the overall number of clay particles remained challenging to estimate.

Characterization of both FWS flocs and aggregates was performed to (1) estimate the size distribution and ESD of FWS and agglomerates, (2) assess the number of MPs in each agglomerate and (3) estimate the FWS and agglomerate density. Individual flocs were gently removed from suspension by pipette, where the end of the 1 ml pipette tip was cut off to increase the diameter of the opening to approximately 3 mm to capture agglomerates without destroying them. Flocs were placed on a glass slide and observed under a fluorescent microscope (LEICA DM4 B; Supplementary Fig. 3). Images were processed with ImageJ to measure the size (ESD) and approximate the number of MPs associated within each agglomerate. Thirty FWS flocs and 15 agglomerates of each polymer and size class were sampled and characterized; representative examples are provided in the Supplementary Information (Supplementary Figs. 2, 3 and 7 and Supplementary Table 1).

There is no unequivocal manner to assess the floc and agglomerates density (*ρ*), as the algae component is in fact a porous media. We thus opted for estimating the density of flocs and agglomerates following the procedure outlined in ref. ^50^, with the following equation that has been largely validated for spherical aggregates:

1 $$\rho ={\rho }_\mathrm{w}+\frac{3\times{\rho }_\mathrm{w}\times{C}_\mathrm{d}\times{U}^{2}}{4g\times\mathrm{ESD}}$$

where *ρ*_w_ is the density of the water (section 2.5), *U* is the average settling velocity measured in the experimental set-up, *g* is the gravity acceleration, ESD is the equivalent spherical diameter calculated with ImageJ and *C*_d_ is the drag coefficient calculated with the following equation (White, 1974):

2 $${C}_\mathrm{d}=\frac{24}{\mathrm{Re}}+\frac{6}{1+{\mathrm{Re}}^{0.5}}+0.4$$

where Re is the Reynolds number (>0.5) calculated as:

3 $$\mathrm{Re}=\frac{\mathrm{ESDU}}{\nu }$$

where *ν* is the kinematic viscosity of water (assumed 10^−6 ^m^2^ s^−1^ at 20 °C). Because the equations are valid for nearly spherical particles, the mostly spherical flocs and agglomerates were employed for the calculation of *U*, leading to an appropriated estimation of the density.

### Settling column design and experimental protocol

The settling experiments were conducted in a plexiglass (1 cm wall thickness) column with a square cross section (internal dimensions 15 × 15 × 150 cm) filled with synthetic freshwater (United States Environmental Protection Agency moderately hard water)^51^ (Fig. 2a). The column was filled by pumping water from two 65 l tanks placed underneath the column and emptied via two valves at the bottom of the column, where water was exchanged every three experimental runs to remove particles that had settled to the bottom. Synthetic freshwater was freshly prepared for each experiment and let to rest overnight to equilibrate to room temperature, which was constant and controlled at 20.07 ± 0.51 °C. The water density was measured through a volume-controlled bottle (pycnometer) and was 998 kg m^−^^3^.

The column was illuminated by a high-power laser light (pulsed green laser, wavelength 527 nm, average output power ∼40 W) from the bottom of the column to acquire bright image sequences of the settling particles. A set of lenses (LINOS Focus-Ronar Lenses semi-cylindrical and round) and a mirror was used to narrow the beam to avoid dispersion of power and to ensure it passed straight through the entire column (Fig. 2b). Experiments were conducted in the dark to avoid possible reflections in the images due to the ambient light.

Two different approaches to introduce particles into the top of the column were used because of the relative differences in fragility of the MPs compared to the FWS and agglomerates. For MPs, particles were kept in suspension in an 80 ml glass bottle with a magnetic stirrer and introduced into the column through a peristaltic pump (tubing diameter 2 mm, pump rate 50 r.p.m.). This ensured a continuous delivery of particles with an approximately constant particle number introduced over time. FWS and agglomerates were delivered using a pipette tip to gently move the flocs/aggregates from the stock suspension and spike them into the top of the column. This approach allowed us to preserve their size and structural integrity.

A magnetic stirrer facing the water surface was used to gently disperse particles across the top of the column to avoid unwanted interactions between nearby particles that could have altered the sedimentation rate. The mild turbulence generated by the stirrer was strong enough to disperse particles without breaking any aggregates. To dissipate the turbulence generated by the magnetic stirrer, a polycarbonate honeycomb filter (thickness 60 mm, pore size 3 mm) was installed 15 cm below the air–water interface. Finally, preliminary tests showed that at high particle input concentrations (for example, >600 particles ml^−1^), particles did not distribute well across the top of the column and formed clusters, altering the settling rate. Thus, throughout all experimental variants, particles concentration in the glass bottle was kept below 100 particles ml^−1^. This entire arrangement ensured that (1) particles were well distributed through the column and (2) no artefacts due to particle spiking or mixing impacted the settling velocity measurements.

Settling particles were tracked using a three-dimensional particle tracking velocimetry method. This method consisted of three-dimensional triangulation and tracking of particles by using a stereoscopic camera system^52–55^. This approach involves two or more synchronized cameras, which allowed us to observe the same particle from multiple perspectives. Our set-up consisted of a multi-camera array and was based on four synchronized, high-speed Eosens cameras (Mikrotron) recording at a resolution of 1,280 × 1,024 pixels at up to 500 frames per second and that stream data on a high-capacity storage system (DVR Express Core 2 by IO Industries).

This approach offered several advantages compared with a single camera. First, particle positions were determined in 3D space as opposed to 2D, avoiding errors in the determination of the sedimentation velocity arising from the missing depth information in a 2D system. Second, ambiguities in the detection and tracking of multiple particles simultaneously present in the field of view could be strongly reduced with our multiple camera views. Third, falling trajectories of complex-shaped particles, such as FWS, were complex and three-dimensional (for example, spiralling trajectories), which could be measured properly. Cameras were focused and calibrated on the same observation volume (6 × 6 × 3 cm^3^), located approximately 75 cm below the air–water interface. This long distance ensured that the particles reached their final velocity (zero acceleration) before crossing the observation volume. The camera system was calibrated by imaging a reference object of known coordinates, which allowed to estimate the position and orientation of the cameras in the laboratory reference system and a set of other calibration parameters such as lens and digital distortion. The simultaneous use of four cameras allowed for the tracking of the three-dimensional falling trajectories of a large number of particles (up to thousands per experimental run), providing robust data to assess particle settling velocities and improving subsequent statistical analysis between experimental replicates. The recording and tracking of particles lasted between 5 and 50 minutes, depending on the particles size and density. The laser beam did not warm up the water in the column, so no flow recirculation was detected. Quantification of particle settling rates was achieved by processing the image sequences through the open-source software openPTV (www.openptv.net), which reconstructed the 3D particle trajectories of each individual particle. The velocity vector was then obtained through differentiation of the 3D particle coordinates along their trajectories. On the basis of this, the mean velocity for an individual particle, measured as the average over the particle trajectory through the observation volume, was computed and subsequently statistics (mean and variance) were calculated using all the particles of a given experiment.

Red optical camera filters were applied to the objectives (Nikon AF Micro-Nikor 60 mm with a focal ratio of f/2.8D) to allow easier detection of the fluorescent MPs and avoid the detection of other smaller particles that were inadvertently introduced into the column (mainly dust), which interfered with assessment of MP settling rates. For tracking FWS settling velocities, the camera filters were removed because the autofluorescence of the algae was lower compared with the MPs and it was possible to distinguish the flocs from the dust due to their larger size. Here a threshold was set to exclude any particles smaller than 150 μm (lower limit of FWS ESD distribution, Supplementary Fig. 7) from the analysis in data postprocessing.

### Validation test of experimental set-up

To assess the validity of the entire experimental design in terms of particle dispersion, image acquisition and data postprocessing, spherical fluorescent PET particles (Cospheric, 1,212 kg m^−^^3^, 220–250 μm) were introduced into the column and the measured velocity was then compared with calculated settling velocities following Stokes’ law^56^:

4 $$u=\frac{1}{18}\frac{(\rho -{\rho }_\mathrm{w})}{\mu }g{d}^{2}$$

where *ρ* is the density of the particles (kg m^−^^3^), *ρ*_w_ is the density of the water (998 kg m^−^^3^), *g* is the gravity acceleration, *d* is the radius of the spherical particles and *μ* is the dynamic viscosity of water (1 × 10^−3^ Pa s at 20 °C). This allowed us to compare the predicted settling velocity with the measured velocity of the model spherical MPs of known size and density.

### Statistical analysis

All statistical analyses were conducted with the software Graphpad (Graphpad Prism, 9.2.0 (332) 2021.07.15). All data distributions were first analysed with a set of normality tests (Anderson–Darling, D’Agostino–Pearson, Shapiro–Wilk, Kolmogorov–Smirnov) to test the potential Gaussian distribution. None of the dataset distributions satisfied the tests, therefore a normal distribution of the data could not be assumed. To assess statistically significant differences of mean values between groups, a non-parametric statistical test (Kruskal–Wallis) was conducted, followed by a Dunn’s post-hoc test for pairwise comparison (*α* < 0.05).

### Supplementary information

Supplementary Information Supplementary Figs. 1–7, Discussion 1 and 2, and Tables 1–3.

## Data Availability

The data that support the findings of this study are available from the authors, and within this article and the Supplementary Information. The main raw data that have been generated for this work, shown in Figs. 3, 4 and 6, as well as Supplementary Figs. 1, 5 and 7, are available via figshare at 10.6084/m9.figshare.25683894 (ref. ^60^). Further enquiries can be directed to the corresponding author.
