# Supplementary material for: Impact of heteroaggregation between microplastics and algae on particle vertical transport
Source: Nat Water. 2024 Jun 13;2(6):541–52. doi: 10.1038/s44221-024-00248-z (PMC11192633; doi:10.1038/s44221-024-00248-z)
Supplement: Supplementary file 1 — Supplementary Figs. 1–7, Discussion 1 and 2, and Tables 1–3. [file 44221_2024_248_MOESM1_ESM.pdf]

nature water

Supplementary information https://doi.org/10.1038/s44221-024-00248-z

Impact of heteroaggregation between microplastics and algae on particle vertical transport

In the format provided by the authors and unedited

Table of Contents

Shape factors analysis 2
FWS flocs microscopy imaging 3
Size distribution and density of FWS and FWS-MPs agglomerates 4
Imaging of agglomerates of MPs and FWS 5
Incorporation of clay to FWS and MPs agglomerates 6
ST1: Improved experimental design allowed for wider tracking 7
MPs size distribution profiles 9
Analysis of MPs ESD 10
Microscopy imaging of MPs test materials of all size classes and polymers 11
ST2: Estimation of the number of MPs incorporated into FWS flocks 12
Size distribution of MPs-FWS agglomerates 14
References 15

Shape factors analysis

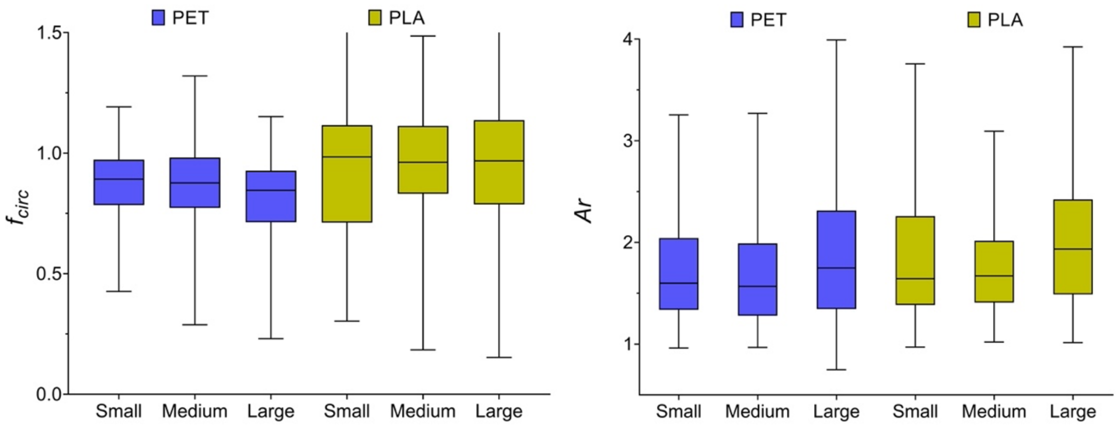

Figure S1: Box plots of circularity (left) and aspect ratio (right) calculations of 100 particles per MPs variant. In each box plot, data are presented within the lower and upper quartile, while the black line indicates the median. The whiskers indicate the upper and lower extremes of the distribution. The circularity of particles indicated how close the fragments were to a sphere. Circularity values are significantly higher for the smaller size of PET compared to large one. No statistically significant differences were found in the profile of the aspect ratio.

Calculations for circularity were performed as follows:

$$f_{circ} = \frac{4\pi A}{P^2} \tag{1}$$

where A is the area of the fragments calculated from the analysis with ImageJ of the microscopy pictures of 100 MPs and P is the perimeter of the equivalent ellipse, which was the best fitting geometry. Although circularity is typically anticipated to fall within the range of 0-1, with 1 representing a perfect circle, certain values shown here approach or exceed unity due to imperfect elliptical geometry fitting, resulting in some values exceeding 1.
The aspect ratio evaluated how large was the length with respect to the width of the fragments, indicating their tendency to be "elongated". It was calculated as follows:

$$A_r = \frac{length}{width} \tag{2}$$

FWS flocs microscopy imaging

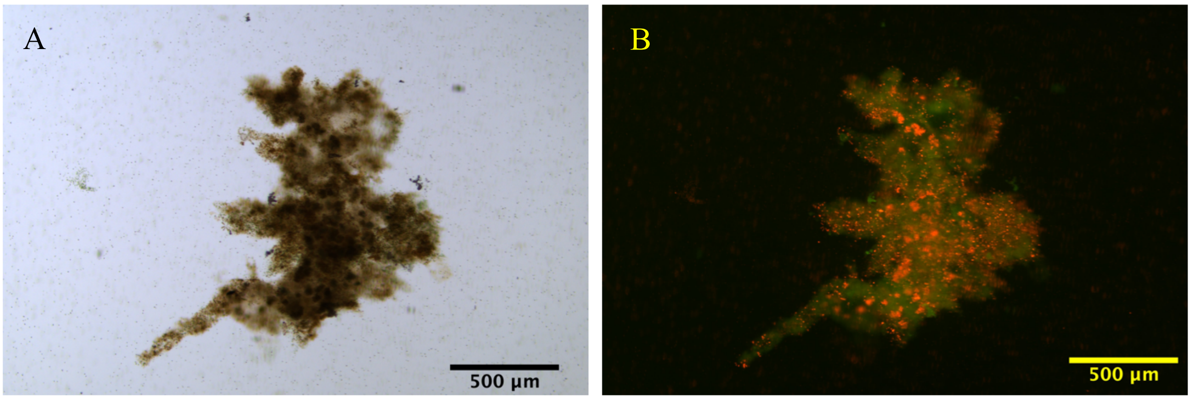

Figure S2: FWS flocks images acquired with LEICA DM4 B in brightfield mode (panel A) and fluorescent mode (panel B). Magnification 5x. Image capture conditions for bright field mode included exposure 36.16, gain 1, saturation 33, intensity 52 while in fluorescent mode was exposure 1.55. The red spots in the fluorescent mode image indicates living cells, while the green ones represent EPS (extracellular polymeric substances) and dead algae cells. Scale bar is 500 µm.

Size distribution and density of FWS and FWS-MPs agglomerates

Table S1: ESD and density of FWS and agglomerates. Values are the average and standard deviation of 30 FWS flocks and 10-15 agglomerates of each polymer and size class couple.

	**PET**				**PP**		**PS**		**FWS**
	Small	Medium	Large	Fibers	Medium	Large	Medium	Large	
**ESD (μm)**	605.66 ± 263.21	904.12 ± 271.21	754.03 ± 233.09	987.58 ± 301.75	509.48 ± 79.70	445.03 ± 79.50	509.86 ± 168.96	354.66 ± 131.82	1161.92 ± 350.18
**Density (kg/m^3)**	1010.52 ± 7.06	1011.259 ± 11.98	1018.66 ± 14.95	1000.80 ± 3.06	1004.75 ± 1.17	1008.81 ± 4.49	1004.06 ± 5.46	1010.25 ± 8.99	1000.19 ± 3.35

Imaging of agglomerates of MPs and FWS

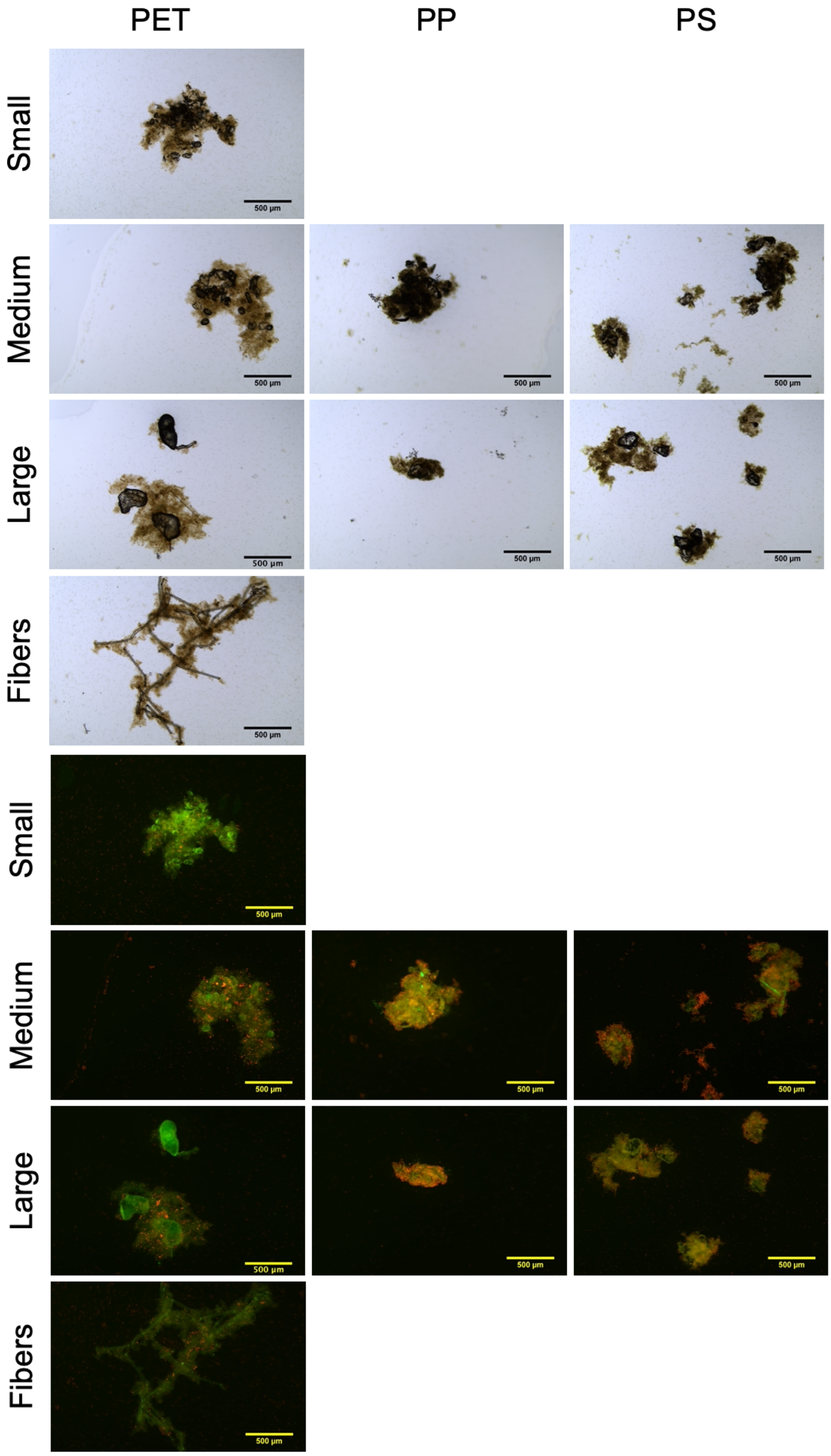

Figure S3: Agglomerates of MPs variants and FWS. Images were acquired with LEICA DM4 B. Magnification 5x. Top set of images: brightfield mode: exposure 36.16, gain 1, saturation 33, intensity 52. Bottom set of images: fluorescent mode: exposure 1.55. Scale bar is 500 µm.

Incorporation of clay to FWS and MPs agglomerates

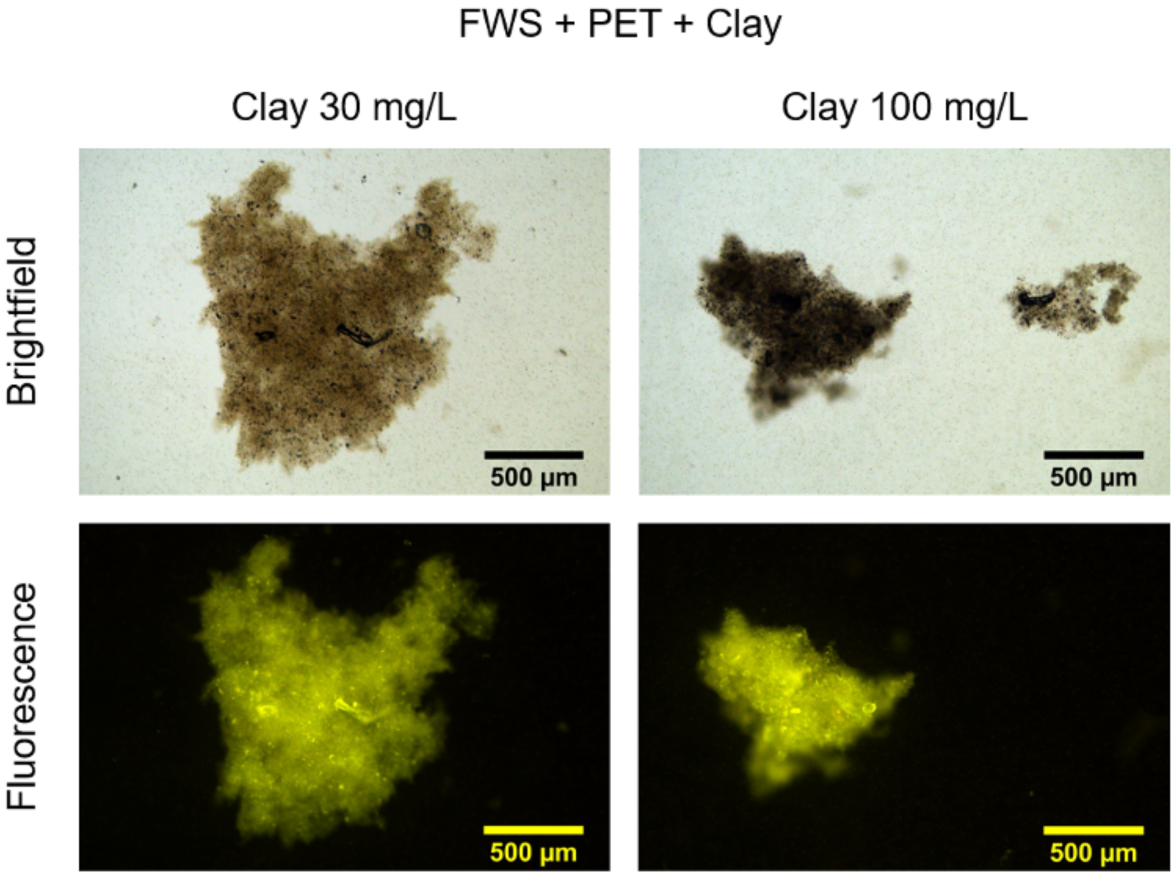

Figure S4: Agglomerates between FWS, PET fragments (size 63-125 µm), with two different clay concentrations (30 and 100 mg/L) in suspension during incubation. The images were acquired with a LEICA DM4 B microscope in both brightfield and fluorescent modes. Image capture conditions for bright field mode included exposure 36.16, gain 1, saturation 33, intensity 52 and in fluorescent mode was exposure 1.55. Magnification 5x. While MPs are still visible in both modes, clay is harder to identify due to its smaller size. Additionally tiny black spots are faintly distinguishable in these flocs compared to those produced without clay (Figure S2 and S3), and their significantly smaller size compared to MPs implies the possibility of these particles being clay homoaggregates incorporated into the flocs. Scale bar is 500 mm.

ST1: Improved experimental design allowed for wider tracking

The methodological improvements developed here, together with considering the interactions between MPs and FWS have on their respective settling rates, has allowed for more robust measurements on how different particle parameters impact settling rates. To date, data collection to quantify particle settling rates has been limited by experimental designs. For MPs, previous approaches included small column systems[1–3] where different introduction methods were used.[4] These methods tested a relatively small number of MPs per experimental run and required longer times to introduce particles into the test system. The experimental design presented in this work made it possible to obtain large amounts of data points. The pump system provided more automation and reduced the time needed to spike MPs in the column, providing robust control on the MPs input concentrations. Particles were evenly spread across the top of the column to avoid clustering and particle-particle interactions, so each individual particle had a constant settling velocity (acceleration equal to zero) when crossing the observation and data collection window. While imaging has previously been used for MPs tracking,[3,4] as only one single camera was used, the number of particles tracked was limited and did not allow for three-dimensional recordings. Using a synchronized and calibrated array of four cameras within the same observation volume, enhanced the overall data collection with the tracking of a greater quantity of particles including the possibility to determine their trajectories over time. Collectively, the experimental design presented here offers a reliable and accessible setup for laboratory measurements, where the simultaneous use of four cameras can track and process thousands of particles per experimental run in a high-throughput fashion.

The column system was also able to measure particles of different morphologies, such as FWS, preserving their fragile structure and providing an accurate alternative to previous in-situ approaches.[5,6] In-situ approaches, such as using SCUBA divers[7,8] or time-lapse cameras,[5,9,10] might face difficulties in terms of sampling conditions, experimental control and repeatability. However, they have the benefit of allowing a direct observation and analysis of natural FWS and MS flocs, which 1) present a heterogenous composition (microbiota, other detritus and suspended matter), 2) are formed over longer time spans and 3) can reach a large sizes (up to 75 mm in maximum length).[7] Creating model snow flocs in the laboratory allows reproducibility with standardized protocols, achieving sizes and morphologies comparable to their natural counterparts.[11,12] However, artificial FWS flocs normally contain simple mixtures of suspended matter (clays, silts, minerals) or microorganisms (algae), which may not completely reflect the heterogeneity of natural snow, which is specific to a defined location and time. Consequently, heteroaggregation between artificial FWS and MPs could be variable compared to natural flocs due to differences in particle physiochemical characteristics. This includes extracellular polymeric substances (EPS) concentration and composition, which can influence surface chemistry interactions between MPs and snow, impacting the number of particles that will be incorporated into the flocs.

Average settling velocities of individual MPs and agglomerate

Table S2: MPs incorporation into FWS agglomerates increased the settling velocity compared to individual MPs. The velocity percentual variation between the velocities of individual MPs and MPs in FWS agglomerates was calculated for each MPs variant.

		Settling velocity (m/s)		Variation (%)
		**MPs**	**MPs in FWS agglomerates**	
**PET**	Small	$3.43 \pm 1.32 \times 10^{-4}$	$19.7 \pm 6.12 \times 10^{-4}$	475
	Medium	$11.9 \pm 7.20 \times 10^{-4}$	$36.3 \pm 16.6 \times 10^{-4}$	203
	Large	$32.9 \pm 13.4 \times 10^{-4}$	$42.1 \pm 15.5 \times 10^{-4}$	28
	Fibers	$4.78 \pm 1.25 \times 10^{-4}$	$11.0 \pm 4.63 \times 10^{-4}$	130
**PLA**	Small	$2.39 \pm 1.00 \times 10^{-4}$	--	--
	Medium	$6.00 \pm 2.17 \times 10^{-4}$	--	--
	Large	$23.4 \pm 11.4 \times 10^{-4}$	--	--
**PP**	Medium	--	$8.84 \pm 0.759 \times 10^{-4}$	--
	Large	--	$10.2 \pm 2.28 \times 10^{-4}$	--
**PS**	Medium	--	$7.00 \pm 2.77 \times 10^{-4}$	--
	Large	--	$8.78 \pm 3.03 \times 10^{-4}$	--
**FWS**	--	$10.3 \pm 6.08 \times 10^{-4}$	--	--

MPs size distribution profiles

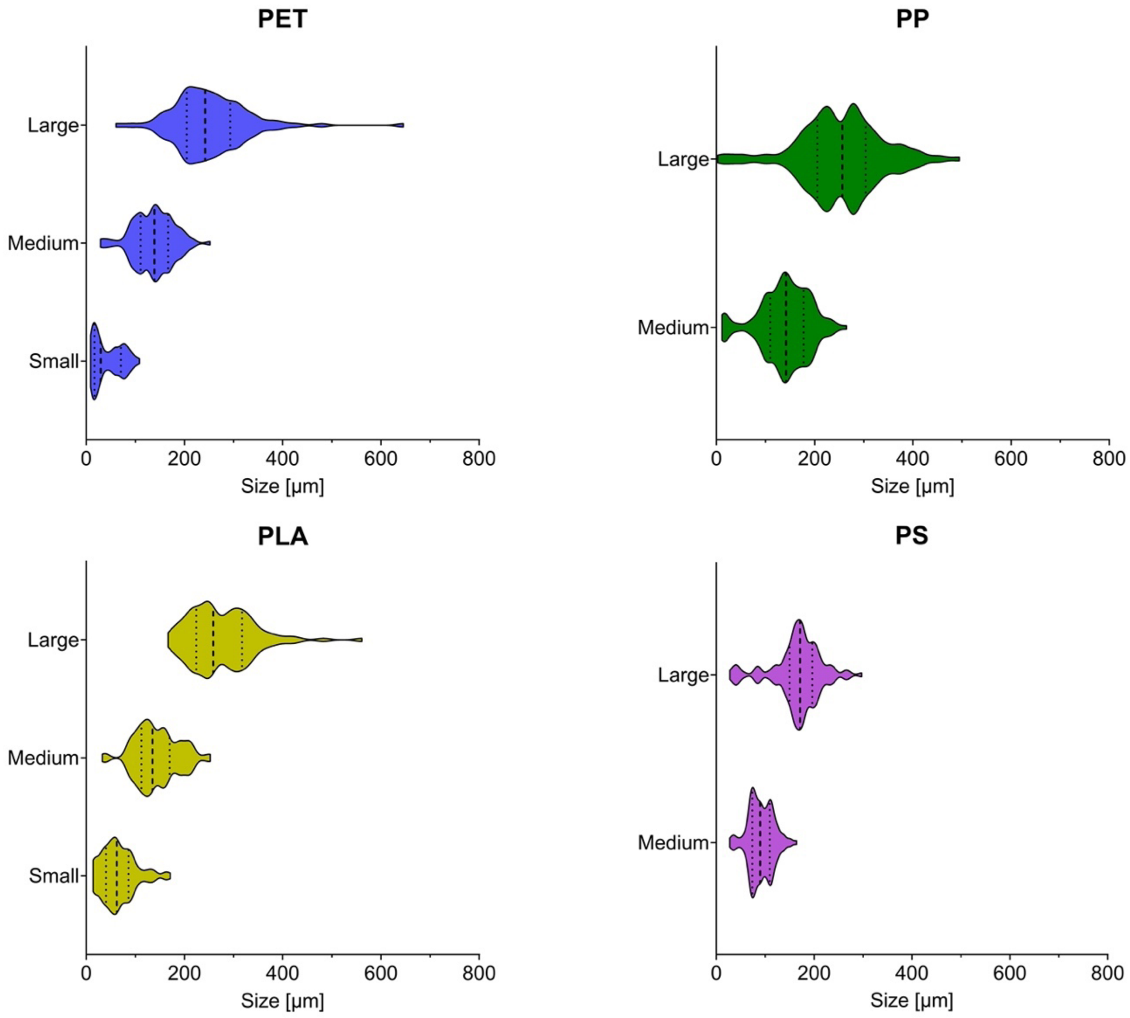

Figure S5: Violin plots of MPs fragments indicating the ESD size distribution. Over 100 particles per MPs variant were analyzed. The thick dotted lines represent the median, while the others indicate quartiles.

Analysis of MPs ESD

Table S3: Average and standard deviation of equivalent spherical diameter (ESD) of MPs fragment classes (at least 100 individual particles measured per variant). MPs ESD were often larger than the nominal sieve size cut off.

	**PET**	**PLA**	**PP**	**PS**
Small<br>< 63 µm	43.8 ± 29.7	66.1 ± 34.4		
Medium<br>63-125 µm	137.0 ± 40.0	143.6 ± 44.0	162.1 ± 35.5	91.4 ± 25.1
Large<br>125-250 µm	253.4 ± 77.6	275.9 ± 66.5	257.6 ± 67.0	166.2 ± 49.2

Microscopy imaging of MPs test materials of all size classes and polymers

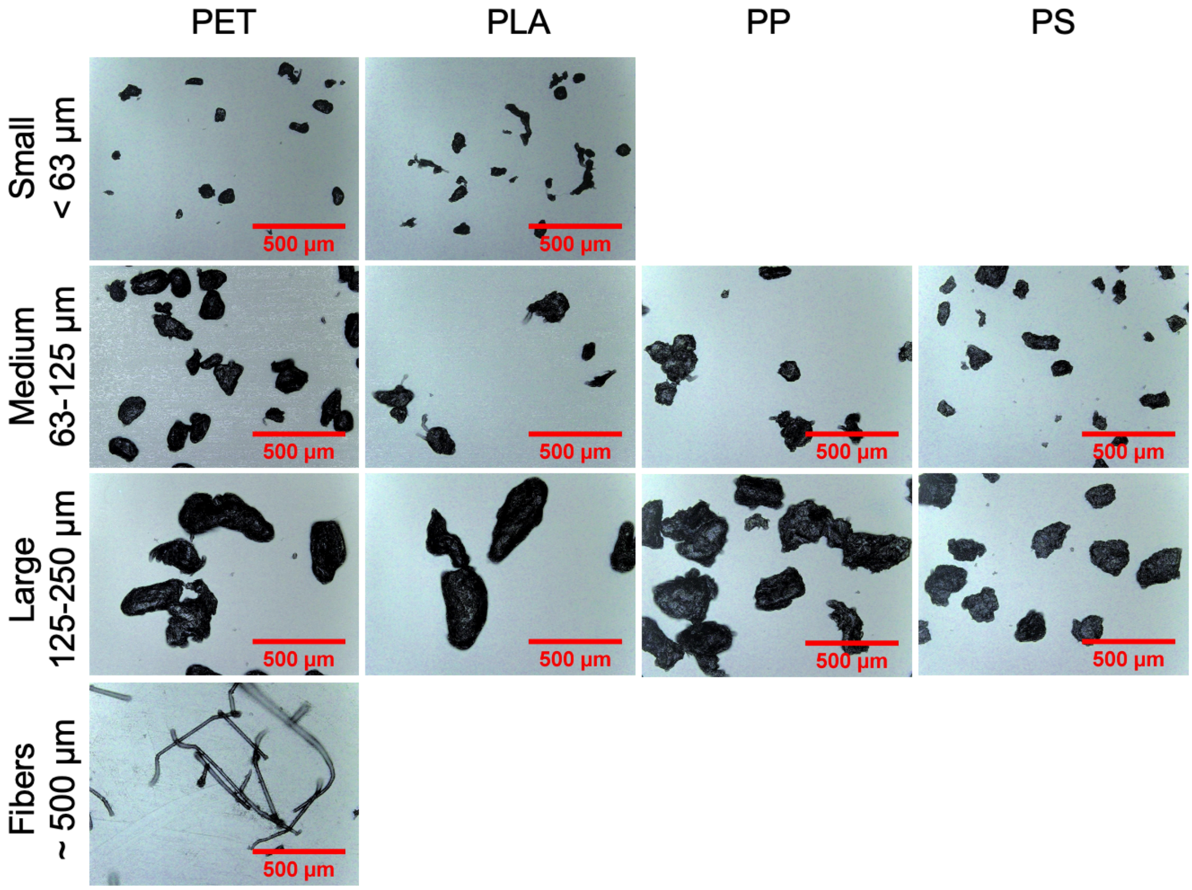

Figure S6: From top row to bottom: small (<63 µm), medium (63-125 µm), large (125-250 µm), fibers (~500 µm length x 30 µm diameter). From left to right: Polyethylene terephthalate (PET, 1.40 g/cm^3), polylactic acid (PLA, 1.25 g/cm^3), polypropylene (PP, 0.90 g/cm^3) and polystyrene (PS, 1.04 g/cm^3). Images were acquired with Keyence 3D Laser Scanning Microscope at 10x magnification. Scale bar is 500 µm.

ST2: Estimation of the number of MPs incorporated into FWS flocks

(1)

The density of a heteroaggregate can be expressed as:

$$\rho_{HA} = (\rho_{FS} * V_{FS} + \rho_P * V_P) * \frac{1}{V_p + V_{FS}}$$

Where ρ_{HA}, ρ_{FS}, ρ_P are the densities of the agglomerates, FWS and MPs, respectively, and V_{FS}, V_P are the volume of the heteroaggregate and MPs. The volume of the heteroaggregate, V_{HA}, can be estimated as follows:

$$V_{HA} = V_P + V_{FS}$$

Substitution into equation (1) yields:

$$\rho_{HA} = \rho_{FS} + \frac{V_P}{V_{HA}}(\rho_P - \rho_{FS}) \qquad (2)$$

The velocity of FWS (u_{FS}) and agglomerates (u_{HA}) in the Stokes' regime can be modelled with a linear dependance on the density and a dependance on the size with a generic function *f(r)*, where *r* is the radius:

$$u_{FS} = (\rho_{FS} - \rho_w) * f(r)$$
$$u_{HA} = (\rho_{HA} - \rho_w) * f(r)$$

where ρw is the density of water (assumed 998 kg/m^3). For example, for a spherical particle $f(r) = 2/9gr^2/\mu$, as in Stokes' law. For other shapes, this function will be more complex. The ratio between the velocity of the agglomerates and FWS of the same radius can then be calculated as:

$$\frac{u_{HA}}{u_{FS}} = \frac{\rho_{HA} - \rho_w}{\rho_{FS} - \rho_w} = 1 + \frac{\rho_P - \rho_{FS}}{\rho_{FS} - \rho_w} * \frac{V_P}{V_{HA}} \qquad (3)$$

For the case of PET MPs ρ_P = 1400 kg/m^3, and the density of FWS (estimated with the method indicated in the main text) is assumed 1000.19 kg/m^3. With these values, expression (3) can be written as:

$$\frac{u_{HA}}{u_{FS}} = 1 + 182 * \frac{V_P}{V_{HA}} \qquad (4)$$

The volume of MPs in the agglomerates, V_P, can be approximated as follows, assuming that particles are spherical:

$$V_P = n_p * \frac{4}{3}\pi r_P^3,$$

where n_p is the number of MPs particles in the HA. The volume of the agglomerates is approximately:

$$V_{HA} = \frac{4}{3}\pi r_{HA}^3$$

The ratio of the two volumes is then calculated as:

$$\frac{V_P}{V_{HA}} = n_P * \left(\frac{D_P}{D_{HA}}\right)^3,$$

where we have used that the ratio of the radii is equal to the ratio of the diameters. Substituting this result into equation (4), we obtain the expression to estimate the number of MPs in the agglomerates:

$$n_P = \left(\frac{u_{HA}}{u_{FS}} - 1\right) * \left(\frac{1}{182}\right) * \left(\frac{D_{HA}}{D_P}\right)^3 \quad (5)$$

Based on our measurements of settling velocities of agglomerates and FWS, Eq (5) allows estimating the number of MPs particles contained in agglomerates.

Size distribution of MPs-FWS agglomerates

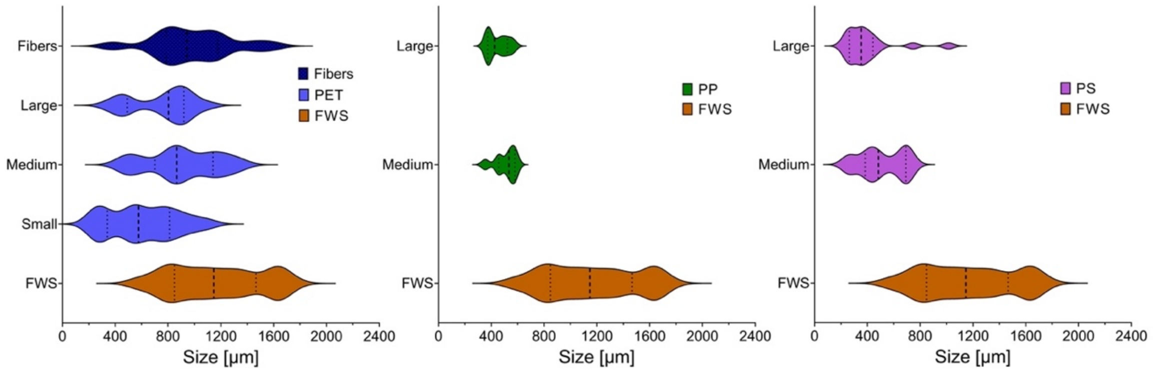

Figure S7: ESD distribution of the agglomerates. FWS alone and agglomerates with PET fragments and fibers (left panel), PP FWS alone and PP agglomerates (center panel) and FWS alone and PS agglomerates (right panel).

References

1. Khatmullina, L. & Isachenko, I. Settling velocity of microplastic particles of regular shapes. *Marine Pollution Bulletin* **114**, 871–880 (2017).
2. Semcesen, P. O. & Wells, M. G. Biofilm growth on buoyant microplastics leads to changes in settling rates: Implications for microplastic retention in the Great Lakes. *Marine Pollution Bulletin* **170**, 112573 (2021).
3. Waldschläger, K. & Schüttrumpf, H. Effects of Particle Properties on the Settling and Rise Velocities of Microplastics in Freshwater under Laboratory Conditions. *Environ. Sci. Technol.* **53**, 1958–1966 (2019).
4. Wang, Z. *et al.* Settling velocity of irregularly shaped microplastics under steady and dynamic flow conditions. *Environ Sci Pollut Res* **28**, 62116–62132 (2021).
5. Lampitt, R. S. Evidence for the seasonal deposition of detritus to the deep-sea floor and its subsequent resuspension. *Deep Sea Research Part A. Oceanographic Research Papers* **32**, 885–897 (1985).
6. Kajihara, M. Settling velocity and porosity of large suspended particle. *Journal of the Oceanographical Society of Japan* **27**, 158–162 (1971).
7. Alldredge, A. L. & Gotschalk, C. In situ settling behavior of marine snow1: Sinking rates of marine snow. *Limnol. Oceanogr.* **33**, 339–351 (1988).
8. Shanks, A. L. & Trent, J. D. Marine snow: sinking rates and potential role in vertical flux. *Deep Sea Research Part A. Oceanographic Research Papers* **27**, 137–143 (1980).
9. Billett, D. S. M., Lampitt, R. S., Rice, A. L. & Mantoura, R. F. C. Seasonal sedimentation of phytoplankton to the deep-sea benthos. *Nature* **302**, 520–522 (1983).
10. Lampitt, R. S., Hillier, W. R. & Challenor, P. G. Seasonal and diel variation in the open ocean concentration of marine snow aggregates. *Nature* **362**, 737–739 (1993).
11. Walch, H., Praetorius, A., von der Kammer, F. & Hofmann, T. Generation of reproducible model freshwater particulate matter analogues to study the interaction with particulate contaminants. *Water Research* **229**, 119385 (2023).
12. Walch, H., von der Kammer, F. & Hofmann, T. Freshwater suspended particulate matter—Key components and processes in floc formation and dynamics. *Water Research* **220**, 118655 (2022).
